# Selective Disruption of the Cerebral Neocortex in Alzheimer's Disease

**DOI:** 10.1371/journal.pone.0012853

**Published:** 2010-09-23

**Authors:** Rahul S. Desikan, Mert R. Sabuncu, Nicholas J. Schmansky, Martin Reuter, Howard J. Cabral, Christopher P. Hess, Michael W. Weiner, Alessandro Biffi, Christopher D. Anderson, Jonathan Rosand, David H. Salat, Thomas L. Kemper, Anders M. Dale, Reisa A. Sperling, Bruce Fischl, the Alzheimer's Disease Neuroimaging Initiative

**Affiliations:** 1 Athinoula A. Martinos Center for Biomedical Imaging, Department of Radiology, Massachusetts General Hospital, Charlestown, Massachusetts, United States of America; 2 Department of Radiology, University of California San Diego, La Jolla, California, United States of America; 3 Department of Biostatistics, Boston University School of Public Health, Boston, Massachusetts, United States of America; 4 Department of Radiology, University of California San Francisco, San Francisco, California, United States of America; 5 Department of Veteran Affairs, San Francisco, California, United States of America; 6 Center for Human Genetic Research, Department of Neurology, Massachusetts General Hospital, Boston, Massachusetts, United States of America; 7 Program in Medical and Population Genetics, Broad Institute, Cambridge, Massachusetts, United States of America; 8 Department of Anatomy and Neurobiology, Boston University School of Medicine, Boston, Massachusetts, United States of America; 9 Department of Neuroscience, University of California San Diego, La Jolla, California, United States of America; 10 Center for Alzheimer Research and Treatment, Department of Neurology, Brigham and Women's Hospital, Boston, Massachusetts, United States of America; 11 Department of Neurology, Massachusetts General Hospital, Boston, Massachusetts, United States of America; 12 Computer Science and Artificial Intelligence Laboratory (CSAIL) and Harvard-MIT Division of Health Sciences and Technology, Massachusetts Institute of Technology, Cambridge, Massachusetts, United States of America; Federal University of Rio de Janeiro, Brazil

## Abstract

**Background:**

Alzheimer's disease (AD) and its transitional state mild cognitive impairment (MCI) are characterized by amyloid plaque and tau neurofibrillary tangle (NFT) deposition within the cerebral neocortex and neuronal loss within the hippocampal formation. However, the precise relationship between pathologic changes in neocortical regions and hippocampal atrophy is largely unknown.

**Methodology/Principal Findings:**

In this study, combining structural MRI scans and automated image analysis tools with reduced cerebrospinal fluid (CSF) Aß levels, a surrogate for intra-cranial amyloid plaques and elevated CSF phosphorylated tau (p-tau) levels, a surrogate for neocortical NFTs, we examined the relationship between the presence of Alzheimer's pathology, gray matter thickness of select neocortical regions, and hippocampal volume in cognitively normal older participants and individuals with MCI and AD (n = 724). Amongst all 3 groups, only select heteromodal cortical regions significantly correlated with hippocampal volume. Amongst MCI and AD individuals, gray matter thickness of the entorhinal cortex and inferior temporal gyrus significantly predicted longitudinal hippocampal volume loss in both amyloid positive and p-tau positive individuals. Amongst cognitively normal older adults, thinning only within the medial portion of the orbital frontal cortex significantly differentiated amyloid positive from amyloid negative individuals whereas thinning only within the entorhinal cortex significantly discriminated p-tau positive from p-tau negative individuals.

**Conclusions/Significance:**

Cortical Aβ and tau pathology affects gray matter thinning within select neocortical regions and potentially contributes to downstream hippocampal degeneration. Neocortical Alzheimer's pathology is evident even amongst older asymptomatic individuals suggesting the existence of a preclinical phase of dementia.

## Introduction

Selective neurodegeneration of the cerebral cortex is a characteristic pathological feature of Alzheimer's disease (AD). Intracellular tau-associated neurofibrillary tangles (NFTs) and extracellular amyloid-ß (Aß) associated plaques show a characteristic laminar and regional pattern of distribution within gray matter, with greater involvement of the medial temporal lobe and heteromodal association areas than primary sensory or motor cortices [1]–[3]. Within heteromodal association cortices, long corticocortical projections and the large pyramidal neurons in layers III and V that give rise to these projections appear to be especially vulnerable [4], [5]. As such, it has been postulated that early disruptions in heteromodal association cortices can contribute to a functional isolation of the hippocampal formation thus giving rise to the clinical hallmark of Alzheimer's disease, progressive memory impairment [6], [7].

Recent advances in neuroimaging and image analysis algorithms allow for the *in vivo* assessment of neuropathologic changes underlying AD. Positron emission tomography (PET) studies examining fibrillar amyloid deposition with Pittsburgh Compound B (PiB) and MRI studies of functional and structural connectivity have observed a significant overlap in a number of neocortical regions that appear preferentially affected in the earliest stages of AD [8]–[14]. Taken collectively, these studies suggest that select heteromodal cortical regions are vulnerable to amyloid deposition and exhibit neuronal dysfunction early in the disease process. In comparison, though several studies have demonstrated a relationship between post-mortem NFT pathology and structural MRI measures of atrophy [15], [16], few have examined *in vivo* the effect of tau burden on atrophy in heteromodal and limbic cortices.

Cortical atrophy resulting from cellular shrinkage, dendritic spine loss, and axonal disruption is reflected as a loss of gray matter that diminishes cortical thickness [17], [18]. Prior MRI studies of AD have shown cortical thinning and gray matter disturbances in medial temporal [19]–[21], temporo-parietal [22]–[25], posterior cingulate and medial frontal cortices [10], [26]. Still, the precise relationship between amyloid and tau pathology, regional neocortical thinning, and hippocampal atrophy is largely unknown. In this study, using significant reductions in cerebrospinal fluid (CSF) Aß levels as a surrogate for elevated intra-cranial amyloid plaques [27], [28] and significant elevations in CSF phosphorylated tau levels as a surrogate for neocortical NFTs [29], we explored relationships between the presence of Alzheimer's pathology, thickness of select neocortical regions and the volume of the hippocampus.

## Methods

### Overview

All 724 participants were selected from the Alzheimer's disease Neuroimaging Initiative (ADNI) database (www.loni.ucla.edu/ADNI). The ADNI is a large multi-site collaborative effort launched in 2003 by the National Institute on Aging, the National Institute of Biomedical Imaging and Bioengineering, the Food and Drug Administration, private pharmaceutical companies and non-profit organizations as a public–private partnership aimed at testing whether serial MRI, PET, other biological markers and clinical and neuropsychological assessment can be combined to measure the progression of MCI and early AD. The Principal Investigator of this initiative is Michael Weiner, MD, and ADNI is the result of many co-investigators from a broad range of academic institutions and private corporations, with subjects recruited from over 50 sites across the US and Canada. For more information, please see http://www.adni-info.org.

### Clinical Assessments and Group Characteristics

Each participant from the ADNI cohort was formally evaluated using eligibility criteria that are described in detail elsewhere (http://www.adni-info.org/index.php?option=com_content&task=view&id=9&Itemid=43). The institutional review boards of all participating institutions approved the procedures for this study. Written informed consent was obtained from all participants or surrogates. Briefly, experienced clinicians conducted independent semi-structured interviews with the participant and a knowledgeable collateral source that included a health history, neurological examination, the Mini-Mental State Examination [30], the CDR-Sum of Boxes [31], and a comprehensive neuropsychological battery.

As illustrated in Table 1, participants from the ADNI database were selected if they were clinically classified as:

- **CN** (n = 208) - Individuals who were cognitively normal at baseline and clinical follow-up (CDR 0).
- **MCI** (n = 353) – Individuals with mild cognitive impairment (MCI) defined using the revised MCI criteria [32].
- **AD** (n = 163) – Individuals who met criteria for probable AD [33] (CDR 1).

**Table 1:** Descriptive statistical information for the participants in the study (means listed with standard deviations in parentheses).

Diagnostic Group	CN	MCI	AD
**Sample Size**	208	353	163
**Age**	76.0(4.9)	74.5(7.4)	74.9(7.5)
**Percent Female**	48%	37%	50%
**Education**	16.1(2.8)	15.7(3.0)	14.8(3.2)
**Mini Mental State Examination**	29.1(1.0)	27.0(1.8)	23.3(1.9)
**CDR-Sum of Boxes**	0	1.6(0.9)	4.2(1.6)
**APOE-ε4 carrier status**	27%	54%	66%
**Mean Hippocampal Volume (head size corrected)**	5.0(0.54)	4.41(0.66)	4.07(0.66)

### MRI image acquisition

All ADNI MRI scans were acquired at multiple sites using either a GE, Siemens, or Philips 1.5T system. Multiple high-resolution T1- weighted volumetric MP-RAGE scans were collected for each subject and the raw DICOM images were downloaded from the public ADNI site (http://www.loni.ucla.edu/ADNI/Data/index.shtml). Parameter values vary depending on scanning site and can be found at http://www.loni.ucla.edu/ADNI/Research/Cores/.

### MRI Image Processing

All MRI scans were processed, with little to no manual intervention using the FreeSurfer software package (version 4.5), freely available at http://surfer.nmr.mgh.harvard.edu. A single MPRAGE MRI acquisition for each participant was normalized for intensity inhomogeneities to create an image volume with high contrast-to-noise [34]. The intensity-normalized volume was used to automatically locate the gray/white matter boundary (white matter surface) [35] and in turn, the gray/CSF boundary (gray matter surface) [36]. Cortical thickness measurements were then obtained by calculating the distance between these two boundaries across the entire cortical mantle of each hemisphere [36].

**Figure 1:**
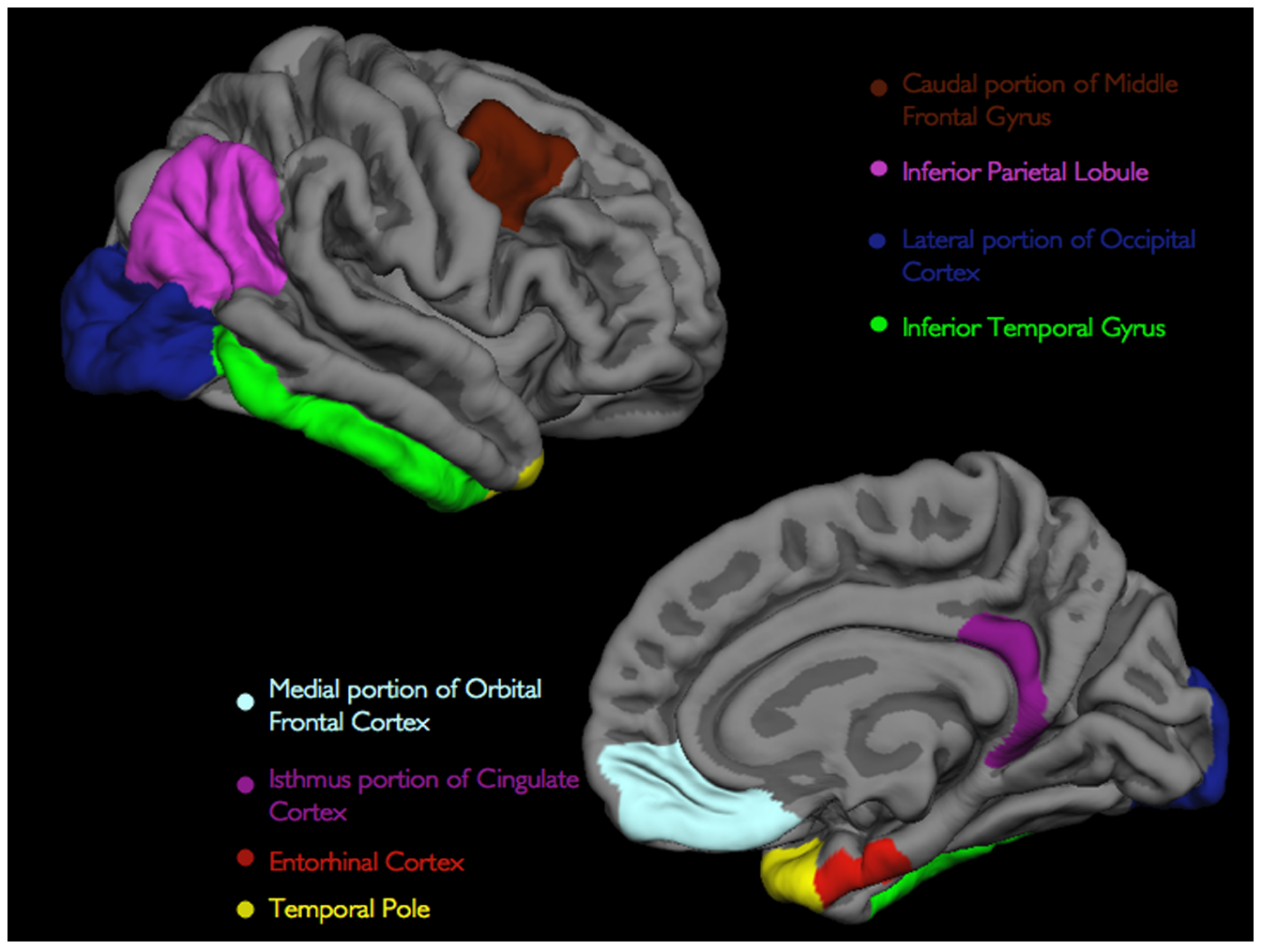
Three-dimensional representations of the 8 ROIs examined in the current study (only right hemisphere is shown). All of the ROIs visible in the lateral (top) and medial (bottom) views of the gray matter surface.

The neocortex of the brain on the MRI scans was then automatically subdivided into gyral-based regions of interest (ROIs). To accomplish this, a registration procedure was used that aligns the cortical folding patterns [37] and probabilistically assigns every point on the cortical surface to one of the 32 ROIs [38]. For the purposes of this study, we focused on 8 ROIs bilaterally corresponding to neocortical regions that prior neuropathological [1]–[3], neuroimaging [19]–[26], and amyloid [10], [12] studies have demonstrated as being affected early in the course of AD. These regions included the 1) caudal portion of the middle frontal gyrus (includes inferior frontal sulcus), 2) medial portion of the orbital frontal cortex, 3) inferior parietal lobule (includes inferior parietal sulcus), 4) lateral portion of the occipital cortex, 5) inferior temporal gyrus, 6) entorhinal cortex, 7) temporal pole, and 8) isthmus portion of the cingulate cortex (includes posterior cingulate and retrosplenial cortices) (Figure 1). The hippocampus was automatically delineated based on an algorithm that uses a probabilistic atlas and examines variations in voxel intensities and spatial relationships to classify subcortical regions on MRI scans [39].

For longitudinal analysis, the baseline and one-year follow-up structural MRI scans were rigid body registered and an unbiased template volume was created [40]. All time points and the template images were then skull stripped, intensity normalized and segmented independently. The longitudinal segmentation results were then obtained by directly mapping the linear Talairach transform and the brain mask from the template to the baseline and follow-up timepoint, and by initializing other processing steps such as the non-linear warps and intensity normalizations with the results from the template. For the baseline and follow-up timepoint, a fused segmentation image that incorporates information of both time points was used as an initial estimate to guide the final delineation of the hippocampus. Atrophy rate of the hippocampus was defined as percent volume loss over the course of one year. A more detailed description of these procedures can be found in [40].

### Cerebrospinal Fluid Measures

From the current ADNI sample, a number of individuals (n = 338) underwent lumbar puncture for CSF biomarker evaluation. Methods for CSF acquisition and biomarker measurement have been reported previously for this sample [28]. In brief, CSF was collected and stored at −80°C at the University of Pennsylvania ADNI Biomarker Core Laboratory. Amyloid beta from peptides 1–42 (Aβ) and tau phosphorylated at threonine 181 (p-tau) was measured using the multiplex xMAP Luminex platform (Luminex Corp, Austin TX) with Innogenetics (INNOBIA AlzBio3, Ghent, Belgium) immunoassay kit–based reagents. Using a previously established CSF-based Aβ cutoff value of 192 pg/ml and a p-tau cutoff value of 23 pg/ml [28], we classified MCI and AD individuals as 1) amyloid positive (n = 193, 43% female, mean age = 74.0, SD = 7.4), 2) amyloid negative (n = 48, 30% female, mean age = 75.9, SD = 8.0), 3) p-tau positive (n = 182, 44% female, mean age = 74.0, SD = 7.5), and 4) p-tau negative (n = 59, 30% female, mean age = 76.1, SD = 7.6). Similarly, we additionally classified CN individuals as a) amyloid positive (n = 35, 54% female, mean age = 76.3, SD = 5.1), b) amyloid negative (n = 61, 50% female, mean age = 75.6, SD = 5.3), c) p-tau positive (n = 34, 47% female, mean age = 77.8, SD = 5.9), and d) p-tau negative (n = 62, 30% female, mean age = 74.7, SD = 4.4). Of note, 46% (n = 16) of the amyloid positive individuals were additionally p-tau positive and 47% (n = 16) of the p-tau positive individuals were additionally amyloid positive.

### Statistical Analysis

Spearman's rank correlation coefficients were first used to examine relationships between the mean thickness of the individual ROIs and total hippocampal volume. Separate linear regression models were constructed for the amyloid positive, amyloid negative, p-tau positive and p-tau negative MCI and AD individuals, with the baseline thickness of the individual ROIs as predictors for hippocampal atrophy rate. Logistic regression models and multivariate analysis of variance (MANOVA) were used to compare whether baseline thickness of the individual ROIs significantly differentiated amyloid positive from amyloid negative as well as p-tau positive from p-tau negative CN individuals. In all of the analyses performed, age, gender, education, and APOE-ε4 carrier status were additionally included.

## Results

### Correlations between regional neocortical thickness and hippocampal volume

Overall, the hippocampus demonstrated the largest magnitude of correlation with the entorhinal cortex (Spearman's ρ = 0.66, p<0.001), temporal pole (Spearman's ρ = 0.51, p<0.001), inferior temporal gyrus (Spearman's ρ = 0.45, p<0.001) and the medial portion of the orbital frontal cortex (Spearman's ρ = 0.41, p<0.001) (Table 2). The inferior parietal lobule and caudal portion of the middle frontal gyrus showed the strongest magnitude of correlation with each other and with the other neocortical regions (Table 2). Within the AD cohort, the hippocampus did not demonstrate any significant correlations with any of the lateral frontal, occipital, or parietal regions including the isthmus portion of the cingulate cortex (Spearman's ρ = 0.12, p>0.05) but correlated significantly with the entorhinal cortex (Spearman's ρ = 0.57, p<0.001), temporal pole (Spearman's ρ = 0.38, p<0.001), inferior temporal gyrus (Spearman's ρ = 0.23, p<0.01), and the medial portion of the orbital frontal cortex (Spearman's ρ = 0.33, p<0.001) (Table 3). In comparison, within MCI cohort, the hippocampus correlated significantly with each neocortical region (Table 4) and similarly, within the CN cohort the hippocampus correlated significantly with all neocortical regions except the lateral portion of the occipital cortex (Spearman's ρ = 0.03, p>0.05) (Table 5).

**Table 2:** Correlation results between cortical thickness of eight neocortical ROIs and total hippocampal volume in all ADNI participants (n = 724).

Regions of Interest	HV	CMF	ERC	IPL	ITG	ISC	LOC	MOF	TP
**HV**	1	0.29	0.66	0.32	0.45	0.36	0.28	0.41	0.51
**CMF**		1	0.38	0.79	0.65	0.60	0.64	0.43	0.42
**ERC**			1	0.43	0.63	0.43	0.36	0.44	0.72
**IPL**				1	0.74	0.67	0.74	0.44	0.44
**ITG**					1	0.63	0.64	0.51	0.64
**ISC**						1	0.52	0.50	0.43
**LOC**							1	0.38	0.38
**MOF**								1	0.48
**TP**									1

Spearman's rank correlation coefficients listed, all p-values significant at <0.0001. All analyses included age, gender, education, and APOE-ε4 carrier status as co-variates. HV = Hippocampal volume, CMF = Caudal portion of middle frontal gyrus thickness, ERC = Entorhinal cortex thickness, IPL = Inferior parietal lobule thickness, ITG = Inferior temporal gyrus thickness, ISC = Isthmus portion of cingulate cortex thickness, LOC = Lateral portion of occipital cortex thickness, MOF = Medial portion of orbital frontal cortex thickness, TP = Temporal pole thickness.

**Table 3:** Correlation results between cortical thickness of eight neocortical ROIs and total hippocampal volume in only AD individuals (n = 163).

Regions of Interest	HV	CMF	ERC	IPL	ITG	ISC	LOC	MOF	TP
**HV**	1	0.02(NS)	0.57(0.0001)	0.03(NS)	0.23(0.003)	0.12(NS)	0.12(NS)	0.33(0.0001)	0.38(0.0001)
**CMF**		1	0.17(0.02)	0.77(0.0001)	0.56(0.0001)	0.58(0.0001)	0.59(0.0001)	0.27(0.0005)	0.24(0.002)
**ERC**			1	0.12(NS)	0.49(0.0001)	0.27(0.007)	0.19(0.01)	0.38(0.0001)	0.67(0.0001)
**IPL**				1	0.63(0.0001)	0.64(0.0001)	0.67(0.0001)	0.24(0.001)	0.23(0.01)
**ITG**					1	0.58(0.0001)	0.51(0.0001)	0.46(0.0001)	0.61(0.0001)
**ISC**						1	0.49(0.0001)	0.42(0.0001)	0.34(0.0001)
**LOC**							1	0.36(0.0001)	0.29(0.0001)
**MOF**								1	0.52(0.0001)
**TP**									1

Spearman's rank correlation coefficients listed with p-values in parentheses. All analyses included age, gender, education, and APOE-ε4 carrier status as co-variates. HV = Hippocampal volume, CMF = Caudal portion of middle frontal gyrus thickness, ERC = Entorhinal cortex thickness, IPL = Inferior parietal lobule thickness, ITG = Inferior temporal gyrus thickness, ISC = Isthmus portion of cingulate cortex thickness, LOC = Lateral portion of occipital cortex thickness, MOF = Medial portion of orbital frontal cortex thickness, TPC = Temporal pole thickness, NS = Not significant.

**Table 4:** Correlation results between cortical thickness of eight neocortical ROIs and total hippocampal volume in only MCI individuals (n = 353).

Regions of Interest	HV	CMF	ERC	IPL	ITG	ISC	LOC	MOF	TP
**HV**	1	0.18	0.61	0.21	0.36	0.28	0.27	0.32	0.48
**CMF**		1	0.27	0.76	0.63	0.57	0.59	0.43	0.34
**ERC**			1	0.31	0.55	0.31	0.30	0.37	0.73
**IPL**				1	0.72	0.65	0.71	0.40	0.37
**ITG**					1	0.58	0.61	0.47	0.59
**ISC**						1	0.48	0.48	0.37
**LOC**							1	0.33	0.31
**MOF**								1	0.43
**TP**									1

Spearman's rank correlation coefficients, all p-values significant at <0.0001. All analyses included age, gender, education, and APOE-ε4 carrier status as co-variates. HV = Hippocampal volume, CMF = Caudal portion of middle frontal gyrus thickness, ERC = Entorhinal cortex thickness, IPL = Inferior parietal lobule thickness, ITG = Inferior temporal gyrus thickness, ISC = Isthmus portion of cingulate cortex thickness, LOC = Lateral portion of occipital cortex thickness, MOF = Medial portion of orbital frontal cortex thickness, TPC = Temporal pole thickness.

**Table 5:** Correlation results between cortical thickness of eight neocortical ROIs and total hippocampal volume in only CN individuals (n = 208).

Regions of Interest	HV	CMF	ERC	IPL	ITG	ISC	LOC	MOF	TP
**HV**	1	0.16(0.02)	0.36(0.0001)	0.15(0.02)	0.20(0.003)	0.19(0.005)	0.03(NS)	0.29(0.0001)	0.23(0.0005)
**CMF**		1	0.27(0.0001)	0.76(0.0001)	0.51(0.0001)	0.49(0.0001)	0.62(0.0001)	0.34(0.0005)	0.38(0.002)
**ERC**			1	0.35(0.0001)	0.52(0.0001)	0.31(0.007)	0.28(0.0001)	0.37(0.0001)	0.56(0.0001)
**IPL**				1	0.63(0.0001)	0.51(0.0001)	0.75(0.0001)	0.38(0.001)	0.40(0.01)
**ITG**					1	0.48(0.0001)	0.57(0.0001)	0.38(0.0001)	0.49(0.0001)
**ISC**						1	0.44(0.0001)	0.38(0.0001)	0.23(0.0005)
**LOC**							1	0.31(0.0001)	0.31(0.0001)
**MOF**								1	0.35(0.0001)
**TP**									1

Spearman's rank correlation coefficients listed with p-values in parentheses. All analyses included age, gender, education, and APOE-ε4 carrier status as co-variates. HV = Hippocampal volume, CMF = Caudal portion of middle frontal gyrus thickness, ERC = Entorhinal cortex thickness, IPL = Inferior parietal lobule thickness, ITG = Inferior temporal gyrus thickness, ISC = Isthmus portion of cingulate cortex thickness, LOC = Lateral portion of occipital cortex thickness, MOF = Medial portion of orbital frontal cortex thickness, TPC = Temporal pole thickness, NS = Not significant.

### Regional cortical thickness, Aβ, and p-tau as predictors of hippocampal atrophy

**Figure 2:**
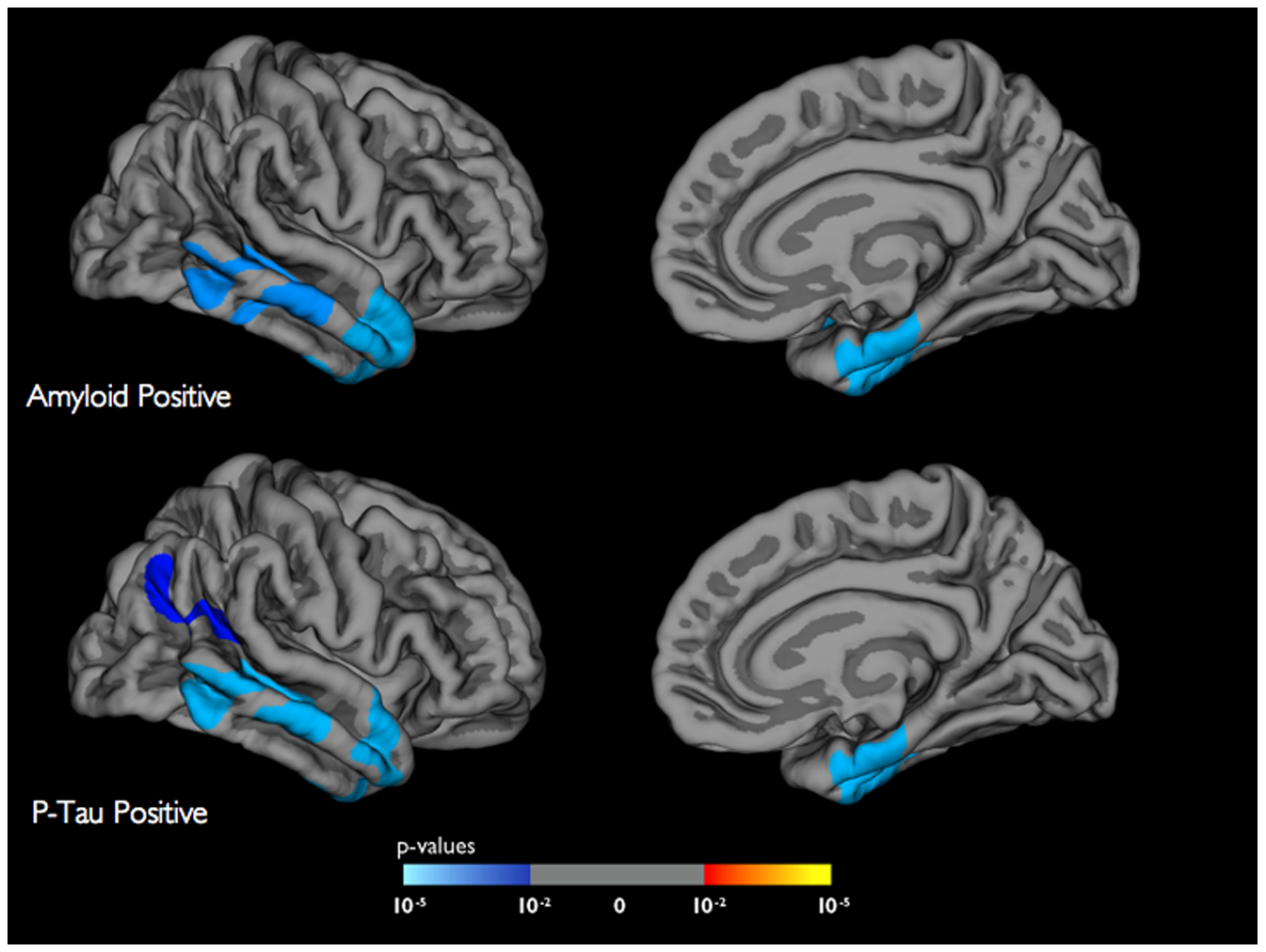
General linear model analyses demonstrating the prediction of longitudinal hippocampal atrophy (over one year) using baseline cortical thickness for the amyloid positive (top panel) and p-tau positive individuals (bottom panel). All results are corrected for multiple comparisons by method of Monte Carlo simulation and shown on the gray matter surface (only right hemisphere). The color scale illustrates the magnitude of effect with blue indicating areas of strongest prediction strength.

Linear regression models amongst the MCI and AD individuals demonstrated that decreased Aβ levels (ß-coefficient = −0.08, 95% Confidence Interval (CI) = −0.01 to −0.003, p<0.01) and increased p-tau levels (ß-coefficient = 0.02, 95% CI = 0.006 to 0.03, p<0.01) significantly predicted hippocampal atrophy rate. Within the amyloid positive MCI and AD individuals, baseline thickness of the entorhinal cortex (ß-coefficient = −0.79, 95% CI = −1.20 to −0.37, p<0.0001) and inferior temporal gyrus (ß-coefficient = −1.04, 95% CI = −2.14 to −0.06, p<0.05) significantly predicted the atrophy rate of the hippocampus. Similarly, within the p-tau positive individuals, baseline thickness of the entorhinal cortex (ß-coefficient = −0.66, 95% CI = −1.09 to −0.23, p<0.01) and inferior temporal gyrus (ß-coefficient = −1.20, 95% CI = −2.31 to −0.10, p<0.05) significantly predicted the atrophy rate of the hippocampus (Figure 2). Within the amyloid negative and p-tau negative MCI and AD individuals, none of the neocortical regions significantly predicted longitudinal hippocampal atrophy.

### Regional cortical thinning in cognitively normal older individuals

**Figure 3:**
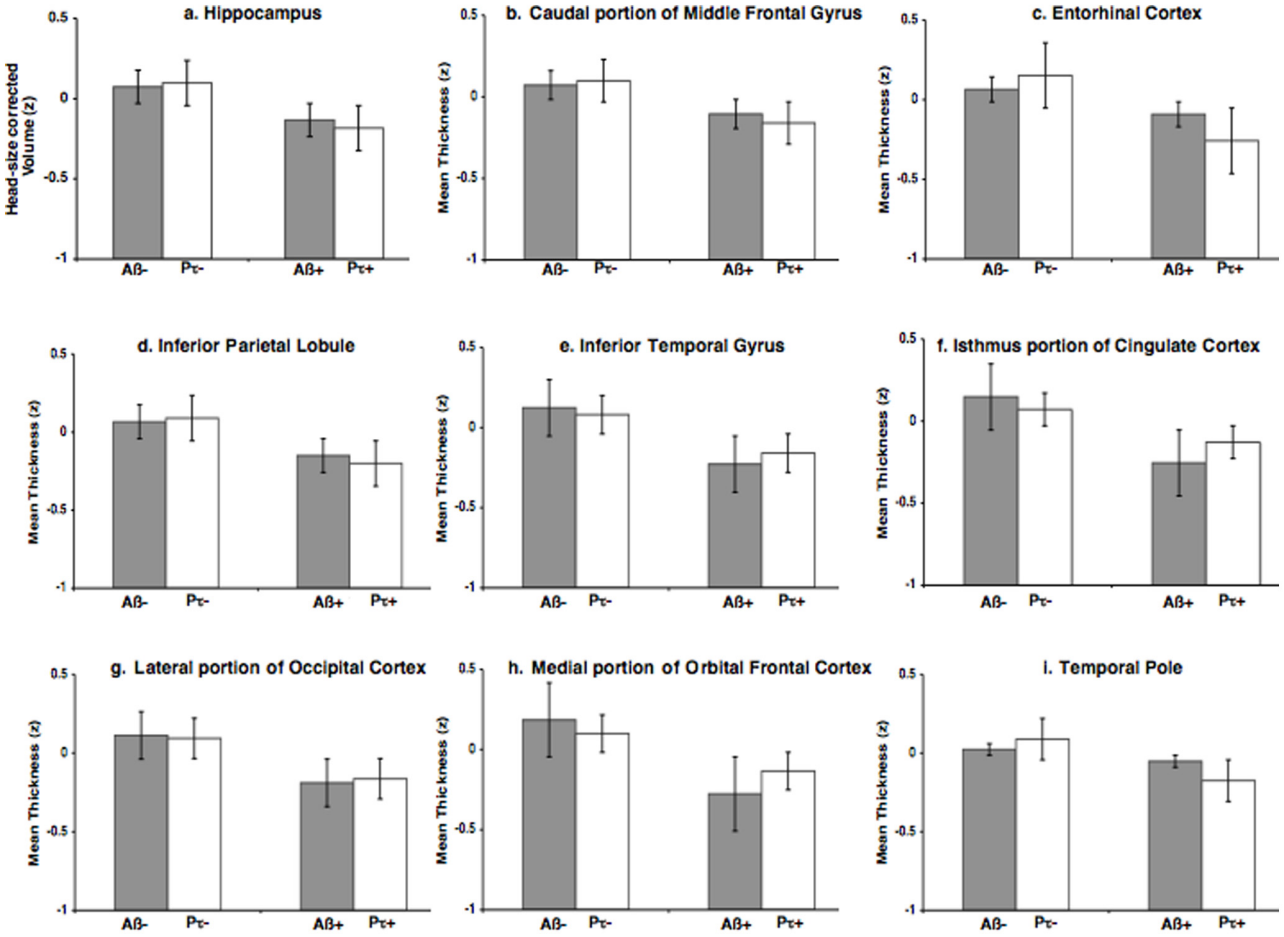
Bar plots illustrating mean cortical thickness, standardized to Z scores, for the 8 neocortical ROIs amongst the amyloid positive (Aβ+), amyloid negative (Aβ−), p-tau positive (Pτ+), and p-tau negative (Pτ−) older individuals. Error bars indicate 1 standard error of the mean.

Logistic regression models amongst the CN cohort demonstrated that APOE-ε4 carrier status (Odds Ratio (OR) = 0.08, 95% CI = 0.02 to 0.27, p<0.0001) and thinning in the medial portion of the orbital frontal cortex (OR = 5.77, 95% CI = 1.12 to 44.80, p<0.05) significantly differentiated the amyloid positive from amyloid negative older individuals whereas thinning of the entorhinal cortex (OR = 2.99, 95% CI = 1.10 to 9.73, p<0.05) significantly differentiated the p-tau positive from p-tau negative older individuals (Figure 3). None of the other measures, including baseline hippocampal volume, showed any significant effects. A MANOVA confirmed that only APOE-ε4 carrier status (F = 18.5, p<0.0001) and thinning of the medial orbital frontal cortex significantly (F = 3.73, p<0.05) differentiated amyloid positive from amyloid negative individuals and only thinning of the entorhinal cortex (F = 3.58, p<0.05) significantly discriminated p-tau positive from p-tau negative individuals.

## Discussion

Our results demonstrate that 1) amongst cognitively normal older participants and individuals with MCI and AD, select heteromodal association cortices correlate with hippocampal volume, 2) amongst MCI and AD individuals, gray matter thinning within the same two neocortical regions predicts longitudinal hippocampal volume loss in both amyloid positive and p-tau positive individuals and 3) amongst cognitively normal older adults, thinning within the medial portion of the orbital frontal cortex significantly differentiates amyloid positive from amyloid negative individuals whereas entorhinal cortex thinning significantly discriminates p-tau positive from p-tau negative individuals. Taken collectively, this indicates that cortical Aβ and tau pathology is evident even among asymptomatic older individuals, affects gray matter thinning within select neocortical regions and potentially contributes to downstream hippocampal degeneration. These findings are in agreement with prior work indicating the value of CSF Aβ as an early biomarker [41] and provide evidence that CSF p-tau and structural MRI measures are additionally informative in the earliest phase of the disease process.

### Relationship between thickness of heteromodal regions and hippocampal volume

The four cerebral cortical regions that best correlated with hippocampal volume follow an interesting neurodevelopmental pattern. On architectonic grounds, the regions identified on the lateral hemisphere trace evolutionary lineage from the so-called ‘paleocortical’ trend whereas regions identified on the medial hemisphere are derived from the ‘archicortical’ trend [42]–[44]. As first described by Kemper [3], these regions collectively subserve the ‘special senses of the head’ including vision, memory, emotion, olfaction, and audition and are inherently heteromodal, binding convergent input from unimodal areas, thus acting as critical gateways for information processing and synthesis [45].

Correlations between thickness of the individual regions and hippocampal volume may reflect underlying patterns of neuropathology. Thickness of the medial portion of the orbital frontal cortex, inferior temporal gyrus, entorhinal cortex, and the temporal pole demonstrated the strongest magnitude of association with the hippocampus amongst each of the three participant groups. In contrast, parietal, occipital, and lateral frontal cortices, correlated with the hippocampus only amongst cognitively normal older adults and MCI individuals. With AD onset, these regions failed to associate with the hippocampus. Of interest, these regions showed the largest magnitude of association with each other amongst each of the three participant groups (Tables 3, 4 and 5).

One explanation for these findings may involve the evolution of the neuropathologic cascade in AD. Amyloid deposits are first noted in the medial portions of the orbital frontal, basal temporal and entorhinal cortices and tau-associated NFTs initially affect the medial temporal and temporopolar cortices [1], [2]. With disease progression, these pathologic changes involve the parietal, primary occipital, and lateral frontal cortices [1]–[3]. Thus, those neocortical regions that show early pathologic changes may correlate with each other throughout the duration of the disease process whereas regions affected later may best associate with each other rather than earlier involved regions.

Another possibility is that associations between regions reflect underlying anatomic connections and the decreased correlation coefficients noted with AD onset represent a selective disruption in cortico-hippocampal connectivity. Though several studies have noted disruptions in anatomic connectivity with AD [8], [13], [46], the fact that the cognitively normal older adults when compared with the MCI and AD individuals demonstrated decreased correlation coefficients between several neocortical regions (including the entorhinal cortex) and the hippocampus (Table 5) suggests that the gray matter associations presented here likely do not represent cortico-hippocampal connectivity. Using diffusion tensor imaging with anatomic localization [47], future work will investigate whether cortico-hippocampal circuitry is selectively vulnerable in dementia.

### Heteromodal cortices, Aβ, and p-tau as predictors of hippocampal atrophy

Significant reductions in CSF Aβ levels and elevations in CSF p-tau levels coupled with thinning within select heteromodal regions predicts longitudinal hippocampal atrophy. Prior PiB studies have demonstrated a significant relationship between neocortical amyloid deposition and hippocampal atrophy [48]–[51]. Our findings indicate that in addition to amyloid, tau may predict downstream hippocampal degeneration. Within both the amyloid and p-tau positive MCI and AD individuals, thickness of the same two neocortical regions (entorhinal cortex and inferior temporal gyrus) (Figure 2) significantly predicted hippocampal atrophy whereas within the amyloid and p-tau negative individuals none of the neocortical regions predicted hippocampal decline. This suggests that those neocortical regions that are likely affected by both Aβ and tau influence hippocampal degeneration.

### Disruption of select heteromodal regions amongst asymptomatic older individuals – evidence for a ‘preclinical’ phase of dementia

Amongst cognitively normal older adults, cortical thinning of the medial orbital frontal cortex significantly differentiated amyloid positive from amyloid negative individuals whereas thinning of the entorhinal cortex significantly discriminated p-tau positive from p-tau negative individuals. Consistent with neuropathology studies, which demonstrate early amyloid deposition within the medial orbital frontal cortex and early NFT accumulation within the entorhinal cortex [1], [2], our results indicate that Aβ and tau pathology differentially affect neocortical regions even among asymptomatic older individuals. To our knowledge, this is the first *in vivo* evidence that amongst cognitively normal individuals tau pathology selectively affects the thickness of the entorhinal cortex. It is important to note that almost half (47%) of the p-tau positive individuals were additionally amyloid positive and future work will involve looking at the interaction between Aβ and tau pathology.

The finding that amyloid positive individuals show selective thinning within the medial orbital frontal cortex is in agreement with one prior study demonstrating that older individuals with subjective memory complaints show significant correlations between gray matter atrophy and neocortical PiB increase within the medial orbital frontal/anterior cingulate cortex [52]. Additionally, this is compatible with functional connectivity studies illustrating that amyloid deposition amongst older individuals disrupts the ‘default network’, which includes the medial aspect of the prefrontal cortex [14], [53]. Consistent with prior work [54], [55], our results indicate that amongst asymptomatic older individuals APOE ε4 carrier status influences CSF Aβ levels but does not affect CSF p-tau levels. Taken together, this indicates that a number of cognitively normal individuals harbor preclinical Alzheimer's pathology and that intracranial Aβ pathology may represent the biological phenotype of the APOE ε4 genotype.

### Clinical Implications and Further Observations

These findings have important clinical implications. Subtle gray matter disturbances in specific regions of the cerebral neocortex are present amongst a subset of asymptomatic older individuals and can be quantified using sensitive structural neuroimaging techniques. In combination with CSF Aβ and tau, structural MRI measures can provide an indication of disease stage and have value in identifying those who will likely benefit from early therapeutic interventions. Our results also indicate the importance of administering anti-amyloid therapy early in the disease process (at a pre-MCI stage) in order to alter or delay hippocampal degeneration and thus progressive memory loss, the harbinger of clinical Alzheimer's dementia.

Why does neurodegeneration affect select cerebral cortical regions? From a phylogenetic perspective, heteromodal and archicortical medial temporal regions constitute the oldest areas of the brain [56], operating as connectional gateways for information synthesis [44], [45], and as such, are likely subject to greater evolutionary selection pressure for degeneration than ‘newer’ brain regions such as the primary sensory areas. Another possibility is that those parts of the brain that need to sustain the highest level of plasticity are most susceptible to degeneration [57]. It is important to note that these two hypotheses need not be considered mutually exclusive. That is, the oldest and densely connected brain regions also presumably have the highest neuroplasticity demands and as such, may be particularly vulnerable to neuropathologic changes.

### Limitations and Caveats

This study has several limitations. One issue involves the use of gyral-based neuroanatomic ROIs to examine specific neocortical regions. It is likely the case that Alzheimer's pathology does not follow the specific boundaries of these ROIs and affects areas within and across multiple ROIs. The use of regions of interest generated from a disease specific effect [19], [20] presents one approach to overcome this limitation. However, as discussed in [58], this method can underestimate or miss disease specific effects potentially leading to false negative findings. Future work will involve combining *a priori* anatomically defined regions with disease-based statistical methods to examine neocortical and subcortical areas in AD. Another limitation involves the use of CSF Aβ and p-tau to assess the presence of amyloid and neurofibrillary pathology in the neocortex. Though amyloid load as measured using PiB and CSF Aβ are highly correlated and likely reflect plaque deposition [59], assessments using PiB may provide a more direct estimate of neocortical amyloid. Similarly, though prior work indicates that CSF p-tau and cortical NFTs are significantly correlated in AD [29], measurements using PET imaging agents that target tangle deposits [60] may offer a more exact estimate of neocortical neurofibrillary pathology.
